# Recent advances in the treatment of multiple myeloma: a brief review

**DOI:** 10.12703/r/11-28

**Published:** 2022-09-29

**Authors:** Arthur Bobin, Xavier Leleu

**Affiliations:** 1 Department of Hematology, University Hospital of Poitiers, 2 rue de la Milétrie, CHU de Poitiers, Poitiers, France

**Keywords:** multiple myeloma, immunotherapy, CD38, CAR-T

## Abstract

The recent history of multiple myeloma has been marked by tremendous advances in the treatments available, which have ultimately improved the patients’ survival. Immune-based therapies, starting with the emergence of anti-CD38 monoclonal antibodies, whose impact is seen across all groups of patients, are probably the greatest evolution in the field of myeloma so far. Building on the efficacy of immunotherapy, “modern” immunological treatments such as CAR-T cells or bispecific antibodies are being developed. There clearly are lots of expectations for these novel immunotherapies, and, though first developed in relapsed myeloma, they will surely challenge the current strategies in early lines of treatment. Immunotherapy, since the development of anti-CD38, is a milestone in the treatment of myeloma and has already led to many paradigm shifts. Nevertheless, myeloma remains an incurable disease and diversified options are still required, notably for heavily pretreated patients. Non-immune-based treatments, which were responsible for most successes previously, are not to be completely abandoned. Novel pathophysiological mechanisms have been unraveled in the past few years, and thus, new targets have been identified, leading to the development of new drugs and new drug classes, such as XPO1 inhibitors and anti-BCL-2. Overall, the future of multiple myeloma is full of possibilities and considerable changes are still expected in the sequencing of treatments in the years to come.

## Introduction

Multiple myeloma (MM), a plasma cell (PC) malignancy, is the second most common hematological malignancy. The treatment of MM has greatly evolved over time, and the recent history of MM has been marked by the advent of multiple novel agents. From conventional regimens, based mainly on chemotherapy, to the emergence of immunotherapy, the treatment options are expanding and, consequently, patients’ survival has improved. Nevertheless, despite the tremendous advances made in the past few years in improving survival, MM remains an incurable disease. Indeed, the drug that would cure MM is still awaited, even if some patients can achieve deep responses and prolonged remissions. We herein summarize the recent therapeutic advances in MM.

## Immune-based treatments: the present and the future of multiple myeloma

The development of immunotherapies is certainly the greatest evolution that has happened in the field of MM recently. Their emergence is a milestone in the history of MM treatment as they have benefited all types of patients, independently of age and setting (upfront vs. relapse). Interestingly, there have been many attempts prior to the era of the anti-CD38 monoclonal antibodies (mAbs) that failed to demonstrate any specific activity in MM. Although anti-CS1/SLAMF7 elotuzumab is the first naïve immunotherapy developed that proved active, it is the anti-CD38 mAbs that have transformed the treatment of MM, now rapidly followed by innovative immunotherapies such as chimeric antigen receptor T (CAR-T) cells and bispecific antibodies (BsAbs). The supremacy of these types of treatment can be explained by a favorable toxicity profile as well as high efficacy. Novel immune-based options with different mechanisms of action, such as antibody-drug conjugates (ADCs), immunomodulators cereblon E3 ligase modulators (CELMoDs), or immune fusion proteins, are still being developed.

### Anti-CD38 monoclonal antibodies

CD38 is a glycoprotein that is highly expressed on malignant PCs. Daratumumab (D), the first-in-class anti-CD38 mAb, is now widely integrated into MM treatment algorithms. The anti-CD38 drug class has been extended with the emergence of isatuximab, which exhibits slightly different mechanisms of action from daratumumab. New anti-CD38 mAbs — like MOR202^1^, which does not activate complement-dependent cytotoxicity mainly responsible for the infusion-related reaction, or TAK-079^2^, which is presented as highly selective of targeted cells, thus reducing off-target adverse events (AEs) — are still surfacing. These last two drugs remain at a very early development stage as of today. The results of the main anti-CD38 phase III trials are presented in Table 1.

**Table 1.** Anti-CD38 monoclonal antibody outcome in multiple myeloma: upfront and relapsed indications.

	ALCYONE (dara-VMP)	MALA (dara-Rd)	CASSIOPEIA (dara-VTd)	POLLUX (dara-Rd)	APOLLO (dara-Pd)	CANDOR (dara-Kd)	ICARIA (isa-Pd)	IKEMA (isa-Kd)
Number of patients	350	368	543	283	151	312	154	179
Median number of prior lines	NA	NA	NA	NA	2	2	3	2
ORR/≥CR, %	90.9/46	92.9/50	/26	92.9/56.6	69/25	84/29	63/5	87
MRD 10^−5^ negativity	28	28.8	64	30.4	9	14	-	30
PFS, months	Median: 36.4	48-month PFS ≈ 60%	48-month PFS ≈ 70%	Median: 44.5	Median: 12.4	18-month PFS ≈ 62%	Median: 11.5	Median: 35.7
OS, months	36-month OS: 78%	-	-	-	-	-	Median: 24.6	-

CR, complete response; MRD, minimal residual disease; NA, not available; ORR, overall response rate; OS, overall survival; PFS, progression-free survival

In the first-line setting for newly diagnosed MM (NDMM), the phase III multicenter CASSIOPEIA trial^3^ demonstrated that the association of D, bortezomib (V), thalidomide (T), and dexamethasone (d) could improve the depth of response and progression-free survival (PFS) in comparison with VTd alone in transplant-eligible (TE) patients. Several other trials are investigating daratumumab and isatuximab in TE patients with NDMM. After the encouraging results of the phase II GRIFFIN trial (42.4% of stringent complete response [CR] rate with D-VRd by the end of consolidation)^4^, the phase III PERSEUS study (ClinicalTrials.gov Identifier: NCT03710603) will further investigate the association with D-VRd but will use the second-generation immunomodulatory drug (IMiD) lenalidomide (R) instead of thalidomide. Indeed, lenalidomide had progressively overtaken thalidomide in recent years and VRd is still a standard of care in most countries worldwide. Data from dara-VRd therefore will be highly anticipated. Isatuximab is also being investigated in two phase 3 studies: isa-VRd (NCT0367731) and isa-KRd (NCT04483739), both for TE NDMM.

Besides, anti-CD38 largely benefited non-transplant-eligible (NTE) patients upfront. The phase III MAIA trial (DRd vs. Rd) led to an impressive median PFS of about 5 years with DRd^5^. For older patients, anti-CD38 mAbs are particularly suitable as they are responsible for limited treatment-related AEs, which allows their administration in the long term. Another option for the treatment of NTE NDMM is the association with D-VMP (M, melphalan; P, prednisone), which showed a median PFS of 36.4 months in the phase III ALCYONE trial^6^. The association with isa-VRd has been tested in the phase 3 IMROZ trial (results are still expected) and in the ongoing BENEFIT/IFM 2020-05 (NCT04751877) with the objective to improve minimal residual disease (MRD) rates. The association of anti-CD38 and VRd for NDMM NTE has also been investigated in the CEPHEUS (dara-VR vs. VRd) study, for which results are also expected.

Overall, anti-CD38 mAbs are finding their way into frontline therapy, especially in Europe, where they are already recommended for nearly all TE and NTE patients, whereas in the US, they tend to be reserved for some selected patients for now. Also, the cost of therapy with anti-CD38 mAbs needs to be addressed.

Before being widely used upfront, anti-CD38 mAbs were used for relapsed or refractory MM (RRMM) as in the CASTOR trial^7^ (DVD; median PFS of 16.7 months, median prior line 2) and the POLLUX trial^8^ (DRd; median PFS of 44.5 months). More recently, the results of the CANDOR trial provided a new option for R-refractory patients, a growing part of patients with RRMM, and the association with DKd led to a median PFS of 28.6 months^9^. Similarly, the APOLLO study^10^ showed a median PFS of 12.4 months for the association of daratumumab and third-generation IMiD pomalidomide (P) (DPd), which can be used in R-exposed/refractory patients. The phase III ICARIA trial demonstrated the activity of isatuximab in association with P and dexamethasone (Isa-Pd) for patients with RRMM (median PFS of 11.5 months, median prior line 3)^11^. Isatuximab in association with proteasome inhibitors represents a promising option for R-refractory patients with RRMM. The phase III IKEMA trial^12^ has investigated the association of isatuximab, K, and dexamethasone (isa-Kd) versus Kd (median PFS of 35.7 months with isa-Kd vs. 19.15 months with Kd, *P* = 0.0007).

The most recent developments with anti-CD38 are based on (i) the objective to combine anti-CD38 with other novel drugs and mechanisms of action to improve the depth of response and particularly the MRD-negative rate and (ii) the focus on MRD to tailor the treatments; in that regard, the phase II MASTER trial provided an interesting induction therapy with D-carfilzomib (K)-Rd (80% MRD 10^−5^ negativity overall and 66% MRD 10^−6^ negativity)^13^. (iii) The question of retreating patients with anti-CD38 mAbs (i.e., sequencing of anti-CD38) is still unanswered, and possibly the new generations of anti-CD38 mAbs will integrate MM treatments only if they display a higher efficacy than daratumumab/isatuximab or if they allow patients to be retreated with the same class. Trials are expected to further explore this hypothesis^14,15^.

### “Armed” immunotherapies

***CAR-T cell therapies*.** CAR-T cells are modified T cells that express a specific antigen (Ag) T-cell receptor that will allow the direct recognition of the Ag without having to go through the major histocompatibility complex. To date, the two most advanced CAR cells are idecabtagene vicleucel (ide-cel/bb2121) and ciltacabtagene autoleucel (cilta-cel), which both target B-cell maturation Ag (BCMA), highly expressed on malignant PCs. Ide-cel was the first CAR-T cell approved in the US and Europe, following the results of the phase II KARMMA 1 trial for patients with advanced RRMM; the overall response rate (ORR) was 73%, 33% of patients had a CR, 18-month PFS was 8.6 months, and median overall survival (OS) was 24.8 months (median follow-up of 15.4 months)^16^. Cilta-cel was evaluated in the phase I/II CARTITUDE-1 study; the ORR was 97.9%, 80.4% of patients had a CR, and 18-month PFS was 66% (median follow-up of 12.4 months)^17^. With CAR-T cells, the main toxicity is the cytokine release syndrome (CRS) that develops after CAR-T cell infusion (median of 1 day with ide-cel and 7 days with cilta-cel studies). CRS occurred in 84% of patients in KARMMA-1 and 95% in CARTITUDE-1, mostly grade 1 or 2. Neurotoxicity (immune effector cell-associated neurotoxicity syndrome, or ICANS) is also a concern with CAR-T cell infusion (18% for ide-cel and 21% for cilta-cel), possibly related to prolonged CRS (would then be cytokine-related), but there are also reports of ICANS related to CAR-T cell expansion (would then be more related to CAR-T cell crossing the brain barrier). Table 2 summarizes the results of KARMMA and CARTITUDE-1.

**Table 2.** CAR-T cells in multiple myeloma: outcome comparison.

	CARTITUDE-1 (cilta-cel)	KARMMA (ide-cel)
Number of patients	97	128
Median number of prior lines	6	6
ORR/≥CR, %	97.9/80.4	73/33
MRD 10^−5^ negativity	93 (n = 57)	26
PFS, months	18-month PFS: 66%	Median: 8.6
Median DOR, months	21.8	10.7
CRS, %	95 4% grade 3/4	84 grade ≥3, 5%
ICANS, %	21 grade ≥3, 10%	18 grade ≥3, 0%

CAR-T, chimeric antigen receptor T; CR, complete response; CRS, cytokine release syndrome; DOR, duration of response; ICANS, immune effector cell-associated neurotoxicity syndrome; MRD, minimal residual disease; ORR, overall response rate; PFS, progression-free survival

Interestingly, CAR-T cells, now reserved for patients with highly previously treated myeloma, are progressively tested earlier in the disease course. For instance, the ongoing phase III CARTITUDE-4 study (cilta-cel vs. VPd or DPd, NCT04181827) will explore CAR-T cells for patients with one to three lines of treatment, similarly to the phase III KARMMA-3 (ide-cel vs. standard regimens, NCT03651128) that will recruit patients who received two to four previous lines.

CAR-T cells are even tested upfront in trials such as CARTITUDE-5 (VRd followed by cilta-cel then Rd for NDMM NTE, NCT04923893) and CARTITUDE-6 (dara-VRd followed by cilta-cel or dara-VRd followed by ASCT, NCT05257083) or as in the KARMMA-4 (NCT04196491) for high-risk NDMM.

There are many developments around the cell therapy CAR concept. In the near future, some progress could still be made with the CAR-T cell therapy (i) by developing allogenic CAR cells (which would make them more rapidly available, “off-the-shelf”), (ii) by selecting T cells that are not in exhaustion and therefore more active, (iii) by looking at CAR natural killer (CAR-NK) cells, and (iv) by moving even further ahead to smoldering MM (sMM).

***Bispecific antibodies*.** BsAbs link functionally active T cells via CD3 and PCs in order to induce T-cell activation and cause lysis of the Ag-expressing target cell. Importantly, the main difference with CAR-T cells is that they are antibody (structure)-based and do not require lymphocytes to be apheresed from the patient and thus can be used “off-the-shelf”, which allows faster delivery. The safety profiles of CAR-T cells and BsAbs are relatively similar as they both lead to an immune reaction of the host, CRS, or neurotoxicity, though to a lesser extent globally. In regard to efficacy, BsAbs are also very active in MM, and no direct comparisons can be run with CAR-T cell therapies.

Currently, two types of constructs exist: bispecific T-cell engagers (BiTes), which lack the Fc fragment and present a short half-life and therefore need to be administered continuously, and “IgG-like” types of molecules with an Fc fragment, which can be delivered intermittently. The first BsAbs have targeted BCMA, similarly to CAR-T cells, and the two most advanced BsAbs are teclistamab (BCMA × CD3) and elranatamab (BCMA × CD3). The results of the first-in-human phase I dose escalation study of teclistamab^18^ showed an ORR of 65% at the recommended phase 2 dose (RP2D) (median follow-up of 6.1 months), and 40% of patients had at least a CR. The most common AEs were mainly CRS (70%) and neutropenia (60%). Elranatamab is another anti-BCMA BsAb and is also under development, the ORR at the RP2D in the phase 1 MagnetisMM-1 study was 83%, and 48 patients (83%) experienced CRS of not more than grade 2.

Moreover, BsAbs in clinical development are targeting new antigens such as GPRC5D (talquetamab) and FcRH5 (cevostamab/BFCR4350A), which are expressed by PCs, or even CD38 (GBR1342). In the first-in-human study of talquetamab^19^, the ORR was 70% at the RP2D (405 µg/kg weekly). Cevostamab has also proven active in RRMM, and the ORR was 54.5% at the 160 mg dose level in the expansion cohort of the phase I study^20^. Preliminary reports show that these BsAbs might be able to rescue progressive disease on anti-BCMA BsAbs in advanced MM.

***Antibody-drug conjugate: belantamab mafodotin*.** ADCs are a class of drugs constituted by a mAb linked to a cytotoxic agent. ADCs will deliver the cytotoxic payload directly into the targeted cells thanks to the binding proprieties of the mAb, so that off-target AEs are theoretically diminished. Belantamab mafodotin (BM) is the first-in-class anti-BCMA ADC, and the mAb is coupled to monomethyl auristatin F (MMAF), the cytotoxic drug, via a protease-resistant linker. BM is an encouraging option for patients whose RRMM has been treated with all the anti-MM drug classes. The phase II DREAMM-2 study introduced BM and led to its approval for RRMM; in that study, the ORR with BM as a single agent was 31% (median PFS of 2.8 months). Notably, the extended 13-month follow-up^21^ showed that the estimated median PFS for responding patients (> very good partial response) was 14 months while median OS for the overall population was 13.7 months. The safety issues are marked principally by the frequent occurrence of corneal keratopathy (all grades, 72%) as well as hematological toxicities (thrombocytopenia, 38%). The use of BM, therefore, requires a careful and regular ophthalmological follow-up. Several DREAMM studies — such as DREAMM-3 (belantamab vs. Pd, phase 3 for registration, NCT04162210), DREAM-8 (belantamab + Pd vs. Pd, NCT04484623), and DREAMM-9 (belantamab + VRd, NCT04091126) — are ongoing or planned.

### Cereblon E3 ligase modulators

CELMoDs are a new IMiD-derived class. Like IMiDs, they bind to the cereblon E3-ubiquitin ligase complex but with a higher affinity to cereblon, thus leading to an increased degradation of IKZF1 and IKZF3, transcription factors essential for MM cell survival. Iberdomide (CC-220) is one of the most advanced CELMoDs so far. The first-in-human multicohort phase I/II trial (CC-220-MM-001) with iberdomide for RRMM determined the RP2D to be 1.6 mg^22^. In the dose expansion phase^23^ of iber + dex, the ORR was 26.2%, median PFS was 3 months, and median OS was 11.2 months (median follow-up of 7.69 months). In this study, iberdomide was also associated with different drugs; the ORRs were 56% for iber-Vd and 45.9% for iber-Dd. Several iberdomide studies are ongoing, especially in the relapse context. For instance, the phase III EXCALIBER trial will explore the association of iber-Dd versus DVd (EudraCT 020-000431-4). Besides, other CELMoD agents such as CC-92480, which was tested in a multicenter phase 1 trial in heavily pretreated patients, are being developed. The ORRs were 21% for the whole cohort and 48% at the therapeutic dose of 1.0 mg. In regard to the safety profile of CELMoDs, the main AEs are hematological, especially marked by a myelosuppression, close to the known IMiD-related safety profile. In the CC-220-MM-001 study, 82.2% of the patients experienced grade 3 or 4 AEs: neutropenia (44.9%), anemia (28%), thrombocytopenia (21.5%), and leukopenia (20.6%). Other AEs included gastrointestinal (GI) disorders (5.6%), fatigue (2.8%), and rash (1.9%).

## Non-immunological agents: the search for novel mechanisms

Non-immunological agents are still needed in the treatment of MM, especially for patients who have received nearly all the drugs available. For that reason, the development of drugs using novel mechanisms or new targets is still encouraged. Among the principal novel agents that emerged in these past years are anti-BCL-2 (venetoclax) and anti-XPO1 (selinexor). Venetoclax represents an effective option for the subgroup of patients exhibiting the t(11;14) translocation, and selinexor is an alternative option for patients with RRMM who have received all the classic drug classes in MM, although it needs close monitoring.

### Venetoclax

Venetoclax is an oral inhibitor of BCL-2 and is already used in several hematological malignancies. The expression of anti-apoptotic BCL-2 proteins is increased in MM, therefore promoting cell survival. The phase III BELLINI trial for patients with RRMM explored the association of venetoclax + Vd versus Vd. The final analysis of the study^24^ (median follow-up of 45.6 months) showed a median PFS of 23.4 months in the venetoclax group versus 11.4 months in the Vd group (*P* = 0.010). Nevertheless, this trial was marked by a high risk of mortality in the venetoclax arm (14 treatment-emergent deaths vs. 1), owing principally to infections (8/14, 57.1%). Notably, the BELLINI trial led to the observation that the higher response rates and longest PFS were for patients who exhibit the t(11;14) translocation, namely the 15 to 20% of patients with MM; median PFS for this subset with Ven-Vd was 36.8 months. That is why venetoclax-based trials are now focusing on such patients. Thus, the phase III CANOVA trial (NCT03539744) is evaluating the association of venetoclax and dexamethasone for RRMM patients presenting with the t(11;14) translocation in comparison with Pd. Therefore, venetoclax could represent the first tailored therapy in MM, and its use will require that t(11;14) translocation status to be assessed. Also, novel BCL-2 inhibitors such as BGB-11417, which is highly selective of BCL-2 and has shown greater anti-tumor activity in xenograft models than venetoclax^25^, are being developed. BGB-11417 is being evaluated in a phase 1b/2 study in MM (NCT04973605).

### Selinexor

Selinexor (S) is a selective inhibitor of nuclear export (SINE) that targets exportin-1 (XPO1). XPO1 is an oncoprotein that mediates the nuclear export and inactivation of several tumor-suppressor proteins. Selinexor was evaluated in the phase IIb STORM trial in association with dexamethasone for penta-exposed patients (R, P, V, K, and D and triple-class-refractory)^26^. The ORR was 26%, median PFS was 3.7 months, and median OS was 8.6 months. Selinexor-based triplets are also under evaluation for patients with RRMM. For instance, the phase III BOSTON trial^27^ (SVd vs. Vd) showed a median PFS of 13.93 months for SVd versus 9.3 months with Vd (*P* = 0.0075). Furthermore, multiple selinexor-based associations (10 associations and 11 arms) are being evaluated in the phase I/II STOMP study^28^. However, there are concerns about the safety profile of selinexor. A retrospective pooled analysis^29^ of the main selinexor^30^ trials (STORM, STOMP, and BOSTON) revealed high rates of non-hematological events such as nausea (68%), decrease in appetite (53%), diarrhea (41%), and vomiting (37%). The use of dexamethasone and prophylactic antiemetics is almost always necessary because of GI toxicity. The major hematological events are thrombocytopenia (66%), which seems to be dose-dependent, and neutropenia (37%). Although its toxicity signature might require careful management, selinexor might still be of interest for patients with RRMM as it provides a novel option with a different mechanism of action.

## Conclusions

The future is bright in MM as many new treatments are emerging year after year. Immunological agents revolutionized the field with their efficacy and their relatively low toxicity profile. In most countries, anti-CD38 can be regarded as the backbone of most associations of treatments, whether for NDMM TE or NTE and even now in sMM. Daratumumab, the first-in-class anti-CD38 mAb, is now followed by the new anti-CD38 generation drug isatuximab (hopefully benefiting patients who have already received this drug class) but has not yet been formally demonstrated in the clinic. The sequencing of anti-CD38 mAbs is being discussed among MM experts and could become a key issue for patients.

The armed immunotherapies (CAR-T cells and BsAbs) surely represent the most promising options, at relapse for heavily pretreated patients for now, and already have been developed in earlier lines. CAR-T cells and BsAbs have their own specificities despite having apparent similar efficacy and safety profiles. They will challenge current standards of care, including autologous stem cell transplant, but might also improve the treatment of subsets of patients having a poor prognosis and not fully benefiting from current treatments, such as high-risk MM. One can expect these armed immunotherapies to eventually replace the drugs we prescribe today upfront and displace these drugs in the relapse setting. Other immunotherapies such as ADCs and CELMoDs, which are basically new immunomodulatory agents, will continue to help the field in sequencing the various immune-based treatments.

On the other hand, the development of non-immune-based treatments will be key for subsets of patients whose tumor cells carry specific abnormalities that could be specifically targeted, such as anti-BCL-2 agents (venetoclax). The anti-BCL-2 agents represent the first biomarker-driven strategy in MM.

In the end, the development of non-immunological drugs must continue as patients will eventually relapse and immunotherapies have not yet allowed a cure for MM to be achieved, even though immune-based treatments seem to be in the pole position to obtain a long remission that could be seen as a functional cure. The identification of new targets is encouraged and expected, as knowledge of MM pathophysiology is increasing, to expand the options of MM treatments to ultimately lead to a cure.
